# Supplementary material for: Dual Monitoring of Cracking and Healing in Self-healing Coatings Using Microcapsules Loaded with Two Fluorescent Dyes
Source: Molecules. 2019 Apr 30;24(9):1679. doi: 10.3390/molecules24091679 (PMC6539550; doi:10.3390/molecules24091679)
Supplement: Supplementary file 1 [file molecules-24-01679-s001.pdf]

Dual Monitoring of Cracking and Healing in Self-healing Coatings using Microcapsules Loaded with Two Fluorescent Dyes

Young Kyu Song [1], Tae Hee Lee [1,2], Jin Chul Kim [1], Kyu Cheol Lee [1], Sang Ho Lee [1] and Seung Man Noh[1], and Young Il Park [1,*]

[1] Research Center for Green Fine Chemicals, Korea Research Institute of Chemical Technology Ulsan 44412, Republic of Korea
[2] Department of Chemical Engineering, Ulsan National Institute of Science and Technology Ulsan 44919, Republic of Korea
* Corresponding authors: Dr. Young Il Park (ypark@krict.re.kr)

Supplementary Materials:

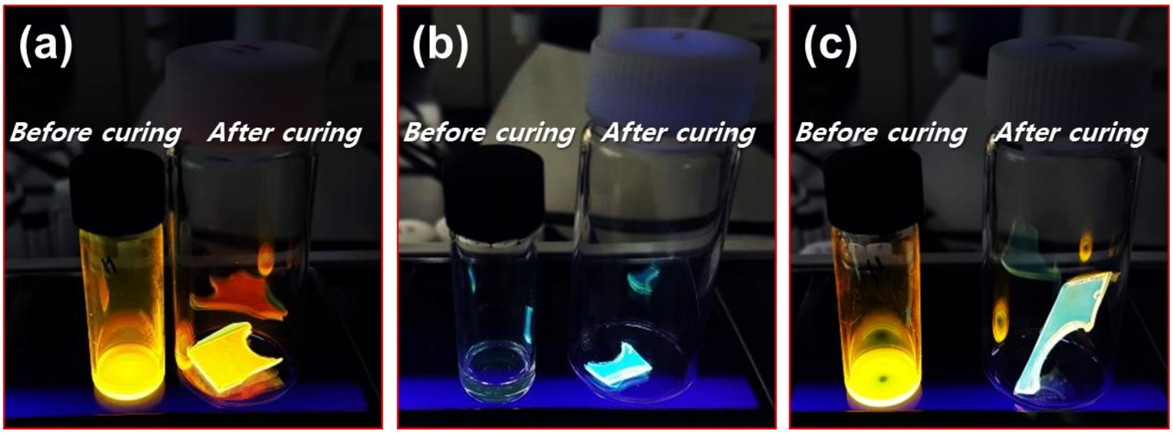

Figure 1. Photographs of (a) normal FL-dye (DCM) (b) AIE-dye (4-TPAE) and (c) mixture of DCM and 4-TPAE in healing agent before and after photo-curing.

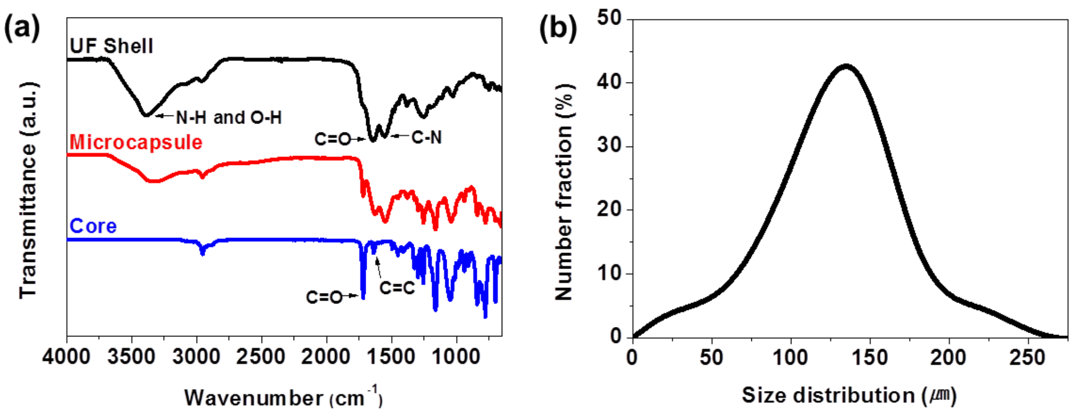

Figure S2. (a) Confirmation of microencapsulation via FT-IR spectral. (b) Size distribution of microcapsule.

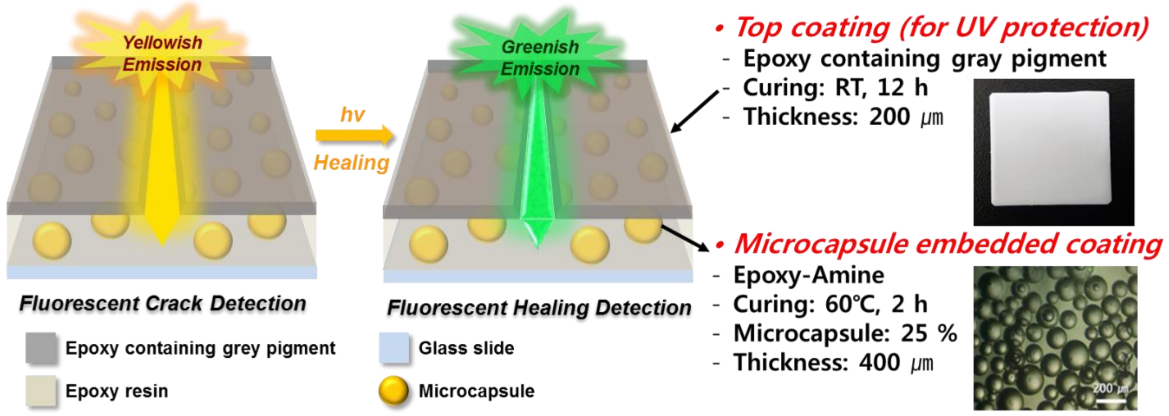

Figure S3. Schematic diagram of self-healing coating system with dye-loaded encapsulate and Figure of top-coating and microcapsule.
